# Dysmenorrhœa—Its Treatment

**Published:** 1878-09

**Authors:** 


DYSMENORRHŒA—ITS TREATMENT.—The practitioner often meets with cases of this disease of a distressing and troublesome type. Numerous remedies and modes of treatment have been proposed, but these often prove inefficient. As this painful and injurious condition may result from different causes, no single plan of treatment will be applicable to every case.

A successful treatment of several obstinate cases prompts us to give the profession the benefit of our plan, which we hope may be deemed worthy of a trial. Believing that constriction or occlusion of the cervix—the result of sub-acute inflammation or displacement—was the cause of the trouble, we have pursued the following method in all cases in which it was not contra-indicated.

About one week before the time for the menstrual flow to commence, we introduce into the cervix a very small tent made from the bark of elm (*ulmus Americanus*). We prefer this material because it is safe and cleanly, and never causes inflammation, as the sponge sometimes does. In most of these cases, we have found it very difficult to pass a small tent, moistened more than half an inch into the cervix, on a first trial, and those used at first are only about one inch in length. After the tent is introduced, a plug of cotton, to which a cord is attached, is passed through the speculum to keep the tent *in situ.* The plug is saturated with carbolic acid and olive oil or glycerin, parts 1 to 7. By means of the cords attached to the tent and plug, the patient removes them the next morning, and uses an anema of warm water and castile soap. In an obstinate case, we use a tent every day on which the flow should commence, unless it is established sooner, substituting longer and stronger ones as the cervical cavity becomes dilated. So much for the mechanical part of our treatment.

According to the indications of the case, we use one of the following remedies internally:

Concentrated tincture of helonias (false unicorn) Keith & Co's.

Fluid extract of ergot (Squibb's).

Tincture of gelsemium.

Syrup of the iodide of iron.

The patient commences taking one of the above at least three weeks before the regular date of her flow, and continues it until this is fully established. She then suspends it for a week or ten days, after which she resumes it. Sometimes we get better results from using two of the above-named remedies alternately, as the helonias and the iron, or the ergot and the iron. A gentle current of electricity is passed through the uterus once a day, for two or three days, before the period. The results of this plan of treatment may be stated briefly, as follows:

During the first period after this treatment, the patient suffers less pain, and the flow is somewhat increased in quantity. If it be persevered in, there will be improvement every month, and after three or four months the cure will most likely be complete.

In all cases of dysmenorrhœa resulting from the causes we have herein set forth, we have found this plan a practical and successful one. The tent used is bland and unirritating, owing to the amount of mucilage it contains, and, by means of the plug, a gentle pressure is kept up against it. As soon as the tent, on removal, is found to be freely stained with blood, we cease to use it until a week before the next period.

This treatment, it will be perceived, is especially adapted to that class of cases in which some eminent practitioners have recommended and practiced incision of the cervix. We vastly prefer the method here described to incision.—*Medical Monthly.*

